# Biological characteristics and pulp regeneration potential of stem cells from canine deciduous teeth compared with those of permanent teeth

**DOI:** 10.1186/s13287-022-03124-3

**Published:** 2022-09-02

**Authors:** S. M. Ziauddin, Misako Nakashima, Hideto Watanabe, Michiyo Tominaga, Koichiro Iohara

**Affiliations:** 1 Regenerative Dental Medicine, National Center for Geriatrics and Gerontology, Research Institute, Geroscience Research Center, 7-430 Morioka, Obu, Aichi 474-8511 Japan; 2 Department of Periodontology and Endodontology, Nagasaki University Graduate, School of Biomedical Sciences, Nagasaki, Japan; 3 Aeras Bio Inc., Air Water Group, Kobe, Hyogo 650-047 Japan; 4 Institute for Molecular Science of Medicine, Aichi Medical University, Nagakute, Aichi 480-1195 Japan

**Keywords:** Deciduous tooth, Permanent tooth, Dental pulp stem cells, Pulp regeneration, Cell bank

## Abstract

**Background:**

Clinical studies have demonstrated that dental pulp stem cells isolated from permanent teeth (PT-DPSCs) are safe and efficacious for complete pulp regeneration in mature pulpectomized permanent teeth with complete apical closure. Moreover, dental pulp stem cells from deciduous teeth (DT-DPSCs) have also been shown to be useful for pulp regenerative cell therapy of injured immature permanent teeth. However, direct comparisons of the pulp regenerative potential of DT-DPSCs and PT-DPSCs from the same individual have not been performed. This study aimed to compare the differences in stem cell properties and pulp regenerative potential of DT-DPSCs and PT-DPSCs of identical origin.

**Methods:**

DT-DPSCs and PT-DPSCs were isolated from the same individual dogs at 4 months and 9 months of age, respectively. The expression of cell surface antigen markers, proliferation and migration activities, and gene expression of stem cell markers, angiogenic/neurotrophic factors and senescence markers were compared. The effects of conditioned medium (CM) derived from these cells on cellular proliferation, migration, angiogenesis, neurite outgrowth and immunosuppression were also compared. Autologous transplantation of DT-DPSCs or PT-DPSCs together with G-CSF was performed to treat pulpectomized teeth in individual dogs. The vascularization and reinnervation of the regenerated pulp tissues were qualitatively and quantitatively compared between groups by histomorphometric analyses.

**Results:**

The rates of positive CXCR4 and G-CSFR expression in DT-DPSCs were significantly higher than those in PT-DPSCs. DT-DPSCs migrated at a higher rate with/without G-CSF and exhibited increased expression of the stem cell markers *Oct3/4* and *CXCR4* and the angiogenic factor VEGF and decreased expression of the senescence marker p16 than PT-DPSCs. DT-DPSC-derived CM promoted increased cell proliferation, migration with G-CSF, and angiogenesis compared with PT-DPSC-derived CM; however, no difference was observed in neurite outgrowth or immunosuppression. The regenerated pulp tissues in the pulpectomized teeth were quantitatively and qualitatively similar between the DT-DPSCs and PT-DPSCs transplant groups.

**Conclusions:**

These results demonstrated that DT-DPSCs could be a potential clinical alternative to PT-DPSCs for pulp regenerative therapy. DT-DPSCs can be preserved in an individual cell bank and used for potential future pulp regenerative therapy before the supply of an individual’s own sound discarded teeth has been exhausted.

**Supplementary Information:**

The online version contains supplementary material available at 10.1186/s13287-022-03124-3.

## Background

Dental pulp is a rich and convenient source of mesenchymal stem cells (MSCs) [1]. Dental pulp stem cells (DPSCs) can be easily isolated from the dental pulp of discarded teeth without raising ethical concerns. In the context of regenerative therapies, DPSCs have great potential for supporting the regeneration of pulp and dentin with remarkable biological properties [2]. We have demonstrated the safety and efficacy of the use of autologous DPSCs derived from permanent teeth for pulp regenerative therapy in permanent mature pulpectomized teeth in preclinical and clinical studies [3, 4]. DPSCs are an optimal cell source; compared with alternative MSCs, such as bone marrow-derived MSCs and adipose-derived MSCs isolated from the same individual dogs and autologously transplanted into the same individuals, DPSCs have higher potential for pulp regeneration, vascularization and reinnervation and lower risk of mineralization in the root canal [5]. Human discarded permanent teeth that are suitable for DPSC isolation are sound teeth without deep caries, and DPSC are conventionally obtained from freshly extracted third molars (wisdom teeth), supernumerary teeth or teeth extracted during orthodontic treatments. However, the limited availability of discarded permanent teeth can be a limitation that must be overcome for the use of autologous DPSCs. Another potential disadvantage of autologous MSCs might be that their stem cell properties are adversely affected by age and by some systemic diseases, such as diabetes and rheumatoid arthritis, which limit their therapeutic potential [6, 7]. Therefore, an alternative source of DPSCs needs to be identified. The stem cells from human exfoliated deciduous teeth (SHEDs) [8] demonstrate very low morbidity, are not associated with ethical concerns regarding their isolation, and are considered candidate progenitor cells due to their intrinsic regenerative capacity [9]. DPSCs isolated from deciduous teeth exhibit higher expression of stem cell markers (Oct3/4) and higher proliferative capacity than DPSCs derived from permanent teeth [9, 10]. A recent clinical trial showed that human autologous DPSCs isolated from deciduous teeth are able to regenerate whole dental pulp with increased root length and reduced apical foramen width in the treatment of injured immature permanent teeth without any adverse events [11].

A case report also demonstrated the effectiveness of allogeneic SHEDs for the treatment of periapical lesions and open apex of immature permanent teeth [12]. Recently, stem cell banking has become popular, and various institutions have established cell banks to facilitate the treatment of challenging diseases and injuries that can occur throughout a lifetime [13]. Replacement of deciduous teeth can create the perfect opportunities to store DPSCs in a stem cell bank [14], which provides a guaranteed donor match for life. Thus, DPSCs isolated from deciduous teeth can be a potential alternative to DPSCs isolated from permanent teeth for the clinical application of pulp regenerative cell therapy to treat permanent mature teeth with complete apical closure.

Our previous study demonstrated that transplanted DPSCs secrete various angiogenic/neurotrophic and immunomodulatory factors without being directly incorporated into vessels, nerves and pulp tissue cells. These paracrine trophic factors are able to induce the migration of endogenous resident cells from the tissue surrounding the teeth into the root canal, inhibit/modulate inflammation and stimulate angiogenesis and reinnervation and G-CSF showed stimulatory effects on migration, antiapoptosis, proliferation, immunosuppression in pulp stem cell culture and DPSCs together with G-CSF enhanced pulp regeneration [3]. Thus, stem cell properties, including strong migratory abilities, strong immunomodulatory/anti-inflammatory effects and enhanced migration and angiogenesis/neurite extension capabilities, might be desirable for pulp regenerative cell therapy. The high proliferative activity and low senescence of DPSCs are also considered beneficial for stem cell banking [15, 16].

DPSCs isolated from deciduous teeth and DPSCs isolated from permanent teeth differ in terms of their immunophenotype and differentiation potential, although they share many biological characteristics [17]. These differences, however, may depend on the inherent heterogeneity of the two populations or different isolation and culture methods [18]. There are no reports that have directly compared the stem cell properties and pulp regeneration efficacy of DPSCs isolated from deciduous teeth and DPSCs isolated from permanent teeth of the same individuals. It is important to clarify whether it is more useful to isolate and culture autologous DPSCs from deciduous teeth before they fall out and then store these DPSCs in a cell bank for future pulp regenerative therapy than to use autologous DPSCs isolated from permanent teeth that are discarded after extraction when root canal treatment is needed. Thus, the present animal model study aimed to examine the difference in the stem cell properties and pulp regenerative potential of DPSCs isolated from deciduous teeth and DPSCs isolated from permanent teeth. The two cell populations were isolated from 4-month-old deciduous teeth and 9-month-old permanent teeth of the same individual dogs and cultured based on an accurate standard operating procedure. Their cell characteristics, including the expression of cell surface antigen markers, proliferation rate, migration activity, and gene expression of stem cell markers, trophic factors and senescence markers, were compared. Pulp regeneration abilities were also quantitatively and qualitatively compared after the autologous transplantation of these cell populations into pulpectomized teeth of the same individual dogs. Our results demonstrated that DPSCs isolated from deciduous teeth could be a potential clinical alternative to DPSCs isolated from permanent teeth for use in pulp regenerative cell therapy.

## Methods

### Culture of DT-DPSCs and PT-DPSCs

This study was approved and conducted in accordance with the guidelines of the Animal Protocol Committees of the National Center for Geriatrics and Gerontology, Research Institute (approval #30-19) and Aichi Medical University (approval #31-17). Young female beagle dogs (*n* = 3, Kitayama Lab, Ina, Japan) were used for DPSC isolation; DT-DPSCs were isolated from maxillary deciduous canines at 4 months of age, and PT-DPSCs were isolated from the permanent maxillary premolars of the same dogs at 9 months of age. DT-DPSCs and PT-DPSCs were cultured under hypoxic conditions in a closed container (Animal Stem Cell, Tokyo, Japan) as described previously [19]. The primary colony-derived DT-DPSCs and PT-DPSCs were expanded up to the 4th passage, and the cells were detached and cryopreserved at 1 × 10^6^ cells/mL in a stem cell banker (ZENOAQ Co., LTD., Fukushima, Japan) for further experiments.

### Flow cytometric analysis

When the DT-DPSCs and PT-DPSCs reached the 4th passage, they were characterized by a FACSAriaTM II flow cytometer (BD Biosciences) as described previously [20]. Briefly, the characteristics of DT-DPSCs were compared with those of PT-DPSCs by staining with antibodies against CD29 (PE-cy7, eBioscience), CD 31 (FITC, BD Bioscience), CD 44 (PE-cy7, eBioscience), CD 105 (PE, BioLegend), CXCR4 (APC, R&D Systems) and G-CSF (FITC, R&D Systems). After incubation for 60 min at 4 °C, the samples were analyzed by a FACS Canto-II flow cytometer (BD Biosciences).

### Doubling time

To measure the population doubling time, the cell number from the 2nd passage to the 3rd passage was calculated by trypan blue staining and using a hemocytometer.

### Odontoblast Differentiation assay

For odontogenic differentiation, DT-DPSCs and PT-DPSCs (3 × 10^5^ cells) were seeded in 6-well cell culture plates (Falcon, Corning, Tewksbury, MA, USA), at 70% confluence, cells were cultured in an odontoblast differentiation medium containing L-ascorbic acid-2-phosphate (50 µg/mL; Sigma-Aldrich, St. Louis, MO, USA), β-glycerophosphate (10 mM; Sigma-Aldrich), and dexamethasone (100 nM; Sigma-Aldrich) for 14 days. Mineral nodules were stained with alizarin red stain (Wako Pure Chemical Industries, Japan) after 4% paraformaldehyde fixation for 10 min at room temperature. The area of each alizarin red staining positive region was observed with a DM-6000B fluorescence microscope (Leica, Germany) and quantified with image J software (version 1.52, imagej.nih.gov).

### Real-time reverse transcription-polymerase chain reaction analysis

Total RNA isolation and real-time RT–PCR were performed as described previously [19]. To examine the mRNA expression of stem cell markers, angiogenic/neurotrophic factors and immunomodulatory markers, real-time PCR analysis was performed with canine-specific primers [Additional file 3: Table S3]. Target gene expression was examined in DT-DPSCs and PT-DPSCs after normalization to β-actin expression.

### Enzyme-linked immunosorbent assay (ELISA)

DT-DPSCs and PT-DPSCs were seeded into 96-well culture plates at a density of 1.0 × 10^5^ cells/well and culture supernatants were collected after 24 h. BDNF and VEGF in culture supernatants were detected using specific enzyme-linked immunosorbent assay (ELISA) kits from R&D systems (Minneapolis, MN, USA). Immunoenzymatic detection was performed according to the protocol described by the manufacturer.

### Migration activity of DT-DPSCs and PT-DPSCs

Migration assay of DT-DPSCs and PT-DPSCs was performed as described previously [19]. Briefly, 1 × 10^5^ DT-DPSCs and PT-DPSCs were incubated in the upper insert of transwell membrane (Corning- Transwell- polycarbonate membrane cell culture inserts, Sigma-Aldrich, Missouri, USA) in 100 µl DMEM. DMEM containing 2% FBS with or without G-CSF (100 ng/ml) was placed in the lower well of 24-well plates. After 24 h incubation, cells on the upper part of insert were removed and the migrating cells in the lower part of insert were fixed with methanol and stained with 1% Giemsa stain for 15 min. After washing cells twice with PBS, stained cells were counted using an inverted bright-field microscope (Leica, 6000B-4, Leica Microsystems GmbH, Wetzlar, Germany) at × 100 magnification.

### The effect of the CM on angiogenesis and neurite extension

When the DT-DPSCs and PT-DPSCs reached 70% confluence, the culture medium was replaced with DMEM, and the CM was collected after 48 h. The CM was concentrated by approximately 25-fold with an Amicon Ultra-15 Centrifugal Filter (Millipore, Billerica, MA, USA). The protein concentration of the CM was measured using a BCA protein assay kit (Pierce, Rockford, IL), and each conditioned medium was used at a final protein concentration of 5 µg/ml. To assess the effect of CM on angiogenesis, human umbilical vein endothelial cells (HUVECs, clone 7F3415, Lonza) were plated on Matrigel (BD Biosciences, San Jose, CA, USA) in DMEM with or without CM, and Endothelial Cell Growth Basal Medium (EBM) (Lonza) supplemented with 5 µg/ml heparin (Lonza, Basel, Switzerland), 5 µg/ml ascorbic acid (Lonza), 5 µg/ml hydrocortisone (Lonza), 5 µg/ml VEGF (Lonza), 5 µg/ml R3-IGF (Lonza), 5 µg/ml hEGF (Lonza), 5 µg/ml GA-1000 (Lonza), and 5 µg/ml hFGF-B (Lonza) was used as a positive control. After 6 h, tube-like structures were observed by an inverted microscope, and the mean length was measured using ImageJ software (version 1.52, imagej.nih.gov).

To examine the effect of CM on neurite extension, human neuroblastoma cells (TGW, clone JCRB 0618, Health Science Research Resources Bank, Japan) were stimulated with DMEM with or without CM, and 50 ng/ml Neurotrophin-3 (Peprotech, London, UK) was used as a positive control. After 24 h, the mean neurite extension length was measured under an inverted microscope using ImageJ software.

### The effect of the combination of CM and G-CSF on migration

The migratory effects of DT-DPSC- and PT-DPSC-derived CM together with G-CSF were compared with those of CM or G-CSF alone in human periodontal ligament fibroblasts [hPdLF 30,315, CC-7049, Lonza (Basel, Switzerland)] as described previously [3]. Briefly, 1 × 10^5^ of hPdLF were plated in the upper insert of transwell membrane in 100 µl DMEM. DMEM containing 2% FBS supplemented with 5 µg/ml CM with or without 100 ng/ml of G-CSF was placed in the lower well of 24-well plates. DMEM with 2% FBS were used as a negative control and G-CSF containing 2% FBS were used as a positive control. After 24 h, the migrating cells were stained as previously described.

### Mixed lymphocyte reactions (MLR) assay

To assess the immunomodulatory effect of CM, a mixed lymphocyte reaction (MLR) assay was performed after collecting canine peripheral blood mononuclear cells (PBMCs) with a BD Vacutainer ® CPT™ Tube (BD, Biosciences). Mitomycin C (Nacalai Tesque, Kyoto, Japan) (final concentration: 10 mg/ml) was used to stimulate allogenic PBMCs for the MLR for 3 h in a humidified 37 °C incubator. Autologous PBMCs and allogenic stimulator PBMCs were cocultured at densities of 5 × 10^4^ cells per well in 96-plates in RPMI-1640 supplemented with 10% FBS (Sigma–Aldrich). In addition, autologous and allogenic stimulator PBMCs treated with 5 μg/ml conditioned medium were added to observe the inhibitory effect on PBMCs growth. After 2 h of incubation, PrestoBlue (Thermo Fisher Scientific, Japan) cell viability reagent was added to each well, and the cell numbers were determined at 0, 12, 24 and 36 h using a spectrophotometer at 450 nm.

### Cell proliferation assay

To assess the proliferative effect of CM, hPdLF were plated at a density of 2.5 × 10^3^ cells per well in 96-well plates with or without 5 µg/ml conditioned medium. After 24, 48 and 72 h, the medium was removed, and 90 µL of normal medium was added to each well and incubated for 30 min at 37 °C in 5% CO_2_. Then, ten microliters of PrestoBlue reagent were added to each well. After 2 h of incubation, fluorescence was measured using a SpectraMax Gemini XPS/EM Plate Reader (Molecular Devices, San Jose, CA, USA) at an excitation wavelength of 535 and an emission wavelength of 615.

### Transplantation of DT-DPSCs and PT-DPSCs into pulpectomized teeth in dogs

Transplantation of DT-DPSCs and PT-DPSCs into the pulpectomized teeth of dogs was performed as described previously [19]. Briefly, upper first and second incisors, a total of 12 teeth, from three 1-year-old young female beagle dogs (Kitayama Lab, Ina, Japan) were used. Transplantation of 5 × 10^5^ DT-DPSCs or PT-DPSCs at together with G-CSF (Neutrogin) in 20 μl of atelocollagen scaffold (1% atelocollagen implant; Koken, Tokyo, Japan) was performed to promote pulp regeneration in the pulpectomized teeth. Four weeks after cell transplantation, the teeth were extracted, and paraffin sections of the regenerated tissues of the teeth were examined by histology. The regenerated tissue was outlined in on-screen image of the histological preparations of each four sections (*n* = 6) by a binocular microscope (Leica, M 205 FA Leica Microsystems, Wetzlar, Germany), and its relative amount to the root canals or morphometric analysis was determined by using Leica Application Suite software (Leica, version 3.4.1). For neovascularization and innervation analyses, Fluorescein Griffonia (Bandeiraea) Simplicifolia Lectin 1/fluorescein-galanthus nivalis (snowdrop) or anti-PGP9.5 (Ultra Clone) (1: 10,000) were used, respectively. The number of odontoblasts, thickness and ratios of newly formed capillary area and neurite extension area to the regenerated pulp area were measured, respectively, by Dynamic cell count BZ-HIC (KEYENCE, Osaka, Japan).

The dogs were observed to monitor clinical symptoms, daily food consumption, and weekly weight change for toxicology assessment. Urinalysis was performed by Clinitek AtlasXL (Sparton Medical Systems, Strongsville, OH, USA) at 4 weeks.

### Statistical analysis

All the data are reported as the mean ± standard deviation (SD). *P* values were calculated using Student’s t test and Tukey’s multiple comparison test in SPSS 25.0 (IBM, Armonk, NY). A *p* value less than 0.05 was considered statistically significant.

## Results

### Flow cytometric analysis of DT-DPSCs and PT-DPSCs

Evaluation of the “stemness” of DT-DPSCs was performed by flow cytometric analysis, and the results were compared with those of PT-DPSCs (Additional file 1: Fig. S1). Both DT-DPSCs and PT-DPSCs were positive for CD29 (99.2 & 97.2%), CD44 (100 & 99.4%), and CD105 (88.4 & 86.7%) expression and negative for CD31 (Table 1) expression, which are minimal criteria for identifying mesenchymal stem cells (MSCs). Notably, the percentages of DT-DPSCs expressing CXCR4 and G-CSFR (26.5 and 60.1%, respectively) were significantly higher than those of PT-DPSCs (21.2 and 39.8%, respectively) (Table 1).

**Table 1:** Expression of cell surface markers in canine deciduous tooth stem cells (DT-DPSCs) compared with canine permanent tooth stem cells (PT-DPSCs)

	DT-DPSCs	PT-DPSCs
CD 29	99.2 ± 0.8	97.2 ± 0.4
CD 31	0.2 ± 0.2	0.3 ± 0.1
CD 44	100 ± 0.0	99.4 ± 0.3
CD 105	88.4 ± 2.0	86.7 ± 2.5
CXCR4	26.5^a^ ± 0.3	21.2 ± 0.4
G-CSFR	60.1^b^ ± 2.8	39.8 ± 0.2

All data are expressed as the means ± SD (*n* = 3). The experiment was repeated three times, and one representative experiment is presented

^a^*p* < 0.05 versus PT-DPSCs

^b^*p* < 0.01 versus PT-DPSCs

### Doubling time of DT-DPSCs and PT-DPSCs

**Fig. 1:**
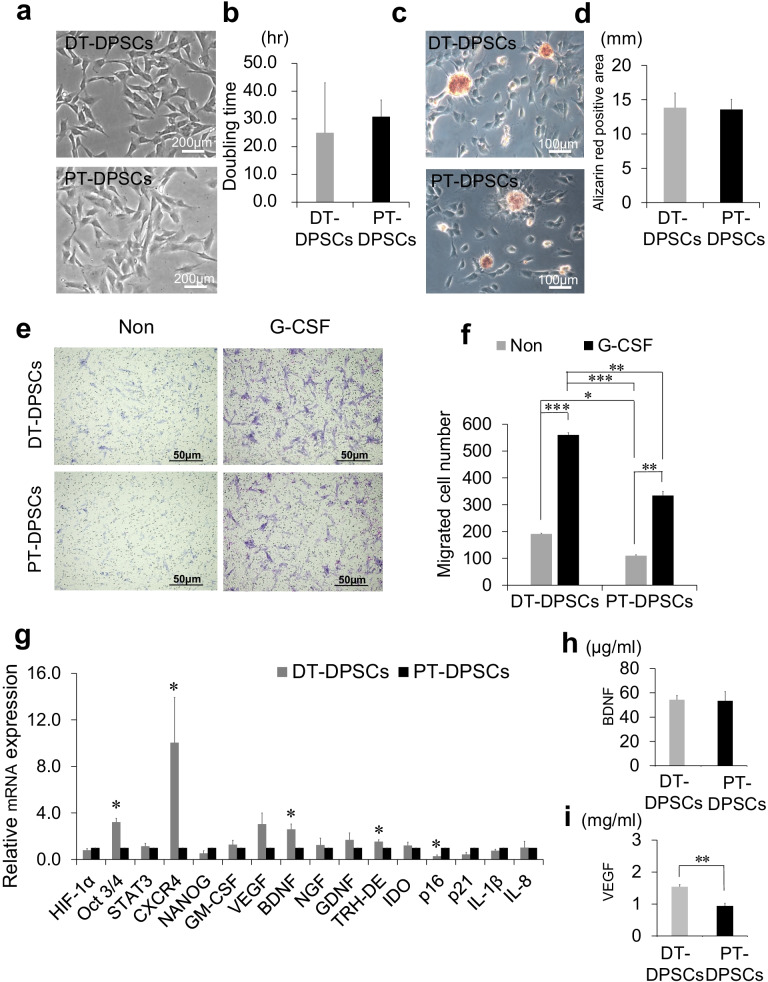
Stem cell properties of DT-DPSCs compared with those of PT-DPSCs. **a** Representative cell morphology at the 3rd passage of culture. **b** Proliferative activity demonstrated by doubling time. **c** Representative images of the Odontoblast differentiation by Alizarin red staining **d** Statistical analysis Alizarin red positive area **e** Migration activity inresponse to G-CSF **f** Migration ability in response to G-CSF were statistically analyzed. *p = 0.03, **p < 0.01, and ***p < 0.001. **g** Gene expression of stem cell markers, angiogenic/neurotrophic factors, pulp tissue markers, immunomodulation markers, senescence markers, and proinflammatory cytokines. Oct 3/4 *p = 0.01, CXCR4 *p = 0.04, BDNF *p = 0.04, TRH-DE *p = 0.01, p 16 *p = 0.03 **h** BDNF expression analyzed by ELISA **i** VEGF expression analyzed by ELISA **p < 0.01. The data are shown as averages of 3 independent experiments. All the data are expressed as the mean ± standard deviation (*n* = 3)

The doubling time of DT-DPSCs was lower than that of PT-DPSCs (Fig. 1b). However, there was no significant difference between the two groups. Additionally, there was no morphological difference between DT-DPSCs and PT-DPSCs (Fig. 1a).

### Odontogenic differentiation ability

The quantification of odontogenesis was identified by the alizarin red staining of calcium phosphate precipitates in DT-DPSCs and PT-DPSCs. DT-DPSCs revealed similar mineral deposition compared to PT-DPSCs (Fig. 1c, d). These results indicate similar odontogenic differentiation potentials between DT-DPSCs and PT-DPSCs.

### Enhanced migratory activity of DT-DPSCs

Compared to PT-DPSCs, DT-DPSCs were demonstrated to exhibit enhanced migratory activity without G-CSF (*p* < 0.05) (Fig. 1e, f). Compared with that of PT-DPSCs, the number of migrating DT-DPSCs was significantly higher in the presence of G-CSF (*p* < 0.01) (Fig. 1e, f). Significant differences were also observed between the group treated with 2% FBS alone as a control and the group treated with 2% FBS together with G-CSF; these differences were observed in DT-DPSCs and PT-DPSCs (*p* < 0.001 and *p* < 0.01, respectively) (Fig. 1f). These results suggested that higher levels of the G-CSF receptor may be expressed by DT-DPSCs than by PT-DPSCs.

### Expression of stem cell markers, trophic factors, senescence-related genes and immunomodulatory genes

The mRNA expression levels of the stem cell markers *Oct3/4* and *CXCR4* (Fig. 1g) were higher in DT-DPSCs than in PT-DPSCs (*p* < 0.05), indicating greater enrichment of stem cell properties. However, other markers, *STAT3* and *NANOG,* were expressed at similar levels between these two cell populations (Fig. 1g). No significant difference in the expression of the angiogenic factors *VEGF* and *GM-CSF* was observed in DT-DPSCs and PT-DPSCs (Fig. 1g). The expression of the neurotrophic factor *BDNF,* but not *GDNF* or *NGF,* was significantly higher in DT-DPSCs than in PT-DPSCs (*p* < 0.05) (Fig. 1g). The expression level of a pulp marker, *TRH-DE*, was higher in DT-DPSCs than in PT-DPSCs (*p* < 0.05); this result suggested greater pulp regenerative potential of DT-DPSCs (Fig. 1g). Furthermore, RT–PCR analysis demonstrated that the expression of the senescence marker *p16* was significantly lower in DT-DPSCs than in PT-DPSCs (*p* < 0.05), but the expression of the immunomodulatory factors *IDO, P21, IL-1β* and *IL-8* and the hypoxic marker *HIF-1a* was similar in DT-DPSCs and PT-DPSCs (Fig. 1g).

### Expression of BDNF and VEGF

ELISA results of cell culture supernatants exhibit similar expression of BDNF in DT-DPSCs and PT-DPSCs (Fig. 1h). However, significantly higher level of VEGF secretion was observed in culture supernatants of DT-DPSCs compared to that of PT-DPSCs (Fig. 1i). These results indicate that DT-DPSCs might have higher angiogenic ability.

### In Vitro Effects of Conditioned Medium

**Fig. 2:**
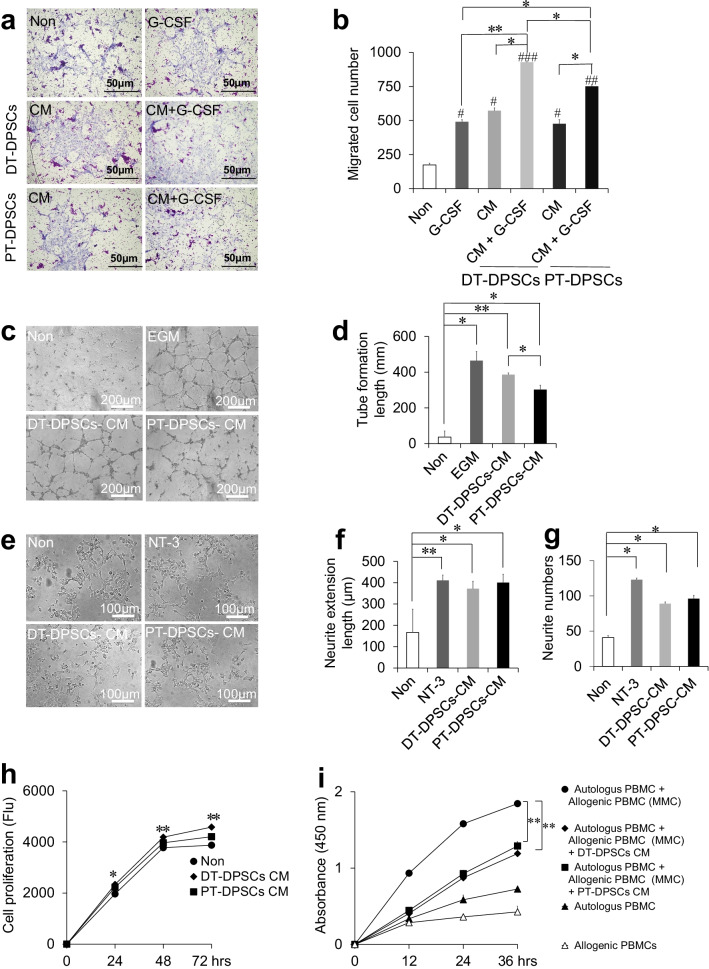
Trophic effects of conditioned medium (CM) from DT-DPSCs compared with that from PT-DPSCs. **a** Migration activity of hPdLF toward the CM with or without G-CSF **b** Enhanced migration of hPdLF toward CM with or without G-CSF after 24 h was statistically analyzed, G-CSF ^#^*p* = 0.04, DT-DPSCs CM ^#^*p* = 0.03 and PT-DPSCs CM ^#^*p* = 0.04 versus non, G-CSF *p = 0.04 versus PT-DPSCs CM + G-CSF, DT-DPSCs CM *p = 0.02 versus DT-DPSCs CM + G-CSF, DT-DPSCs CM + G-CSF *p = 0.04 versus PT-DPSCs CM + G-CSF, PT-DPSCs CM *p = 0.04 versus PT-DPSCs CM + G-CSF, G-CSF versus DT-DPSCs CM + G-CSF **p < 0.01, ^##^*p* < 0.01 and.^###^*p* < 0.001 versus non. **c** Enhanced angiogenic activity of HUVECs, showing network formation after 6 h. **d** Statistical analysis of total tube length. EGM *p = 0.01, PT-DPSCs CM *p = 0.04 and DT-DPSCs CM **p = 0.002 versus Non. DT-DPSCs CM *p = 0.02 versus PT-DPSCs CM. **e** Enhanced neurite outgrowth of the TGW cell line. **f** Statistical analysis of neurite length. DT-DPSCs CM *p = 0.02, PT-DPSCs CM *p = 0.01 versus Non and **p = 0.008. **g** Statistical analysis of neurite numbers. NT-3 *p = 0.02, DT-DPSCs CM *p = 0.03, PT-DPSCs CM *p = 0.04 versus Non **h** Enhanced effect of CM on the proliferative activity of hPdLF. *p = 0.015, **p < 0.01. **i** Mixed lymphocyte reaction (MLR) assay (MMC, treated with mitomycin C) with PBMCs after 24 h. **p < 0.01. All the data are expressed as the mean ± standard deviation (*n* = 3)

We next examined the effect of DT-DPSC- and PT-DPSC-derived CM together with G-CSF on migration ability of hPdLF (Fig. 2a). CM with G-CSF promoted greater migration than CM alone (*p* < 0.05) (Fig. 2b). DT-DPSC-derived CM with G-CSF resulted in higher migratory abilities (*p* < 0.01), and PT-DPSC-derived CM with G-CSF also resulted in higher but less significant (*p* < 0.05) migration than G-CSF alone (Fig. 2b). The number of migrated cells was higher after incubation with DT-DPSC-derived CM than after incubation with PT-DPSC-derived CM; however, no significant difference between the hPdLF incubated with DT-DPSC- and PT-DPSC-derived CM was observed. The migratory abilities of hPdLF incubated with DT-DPSC-derived CM with G-CSF was higher (*p* < 0.05) than those of hPdLF incubated with PT-DPSC-derived CM with G-CSF (Fig. 2b). These results demonstrated the combination of DT-DPSC-derived CM with G-CSF exerted a stronger effect than the combination of PT-DPSC- derived CM with G-CSF.

We further analyzed the effect of DT-DPSC- and PT-DPSC-derived CM on angiogenic and neurite extension abilities. DT-DPSC-derived CM exerted a significantly stronger stimulatory effect on angiogenic tube formation than PT-DPSC-derived CM (*p* < 0.05), and both DT-DPSC- and PT-DPSC- CM promoted significantly stronger (*p* < 0.01 and *p* < 0.05, respectively) angiogenesis compared with the control medium (Fig. 2c, d). These results indicate that the angiogenic potential of DT-DPSCs was better than that of PT-DPSCs and that DT-DPSCs may promote angiogenesis more rapidly in stem cell-mediated dental pulp regeneration. The effects of DT-DPSC- and PT-DPSC-derived CM on neurite outgrowth/neurogenesis were significantly stronger than those of the control medium (Fig. 2e, f), but no significant difference in neurite outgrowth and neurite numbers was observed between human neuroblastoma TGW cells incubated with DT-DPSC- or PT-DPSC-derived CM (Fig. 2f, g).

To examine the effect of DT-DPSC- and PT-DPSC-derived CM on cellular proliferation, the proliferation rate was evaluated by a PrestoBlue cell viability reagent (Fig. 2h). The proliferation assay showed that DT-DPSC-derived CM significantly increased the proliferative capacity of hPdLF when compared to the non-CM control after 24 (*p* < 0.05), 48 and 72 h (*p* < 0.01). These results suggest that DT-DPSC-derived CM enhances cellular proliferation more rapidly than PT-DPSC-derived CM.

In the mixed lymphocyte reaction assay, DT-DPSC- and PT-DPSC-derived CM induced significant reductions in immunosuppression at 36 h compared to non-CM control (*p* < 0.01) (Fig. 2i). No significant difference was observed in immunosuppression in the groups incubated with DT-DPSC- or PT-DPSC-derived CM. These results suggest that the in vitro trophic effects of DT-DPSC-derived CM are similar to those of PT-DPSC-derived CM, leading to high immunosuppressive and immunomodulatory properties.

### Similar Pulp regenerative potential

**Fig. 3:**
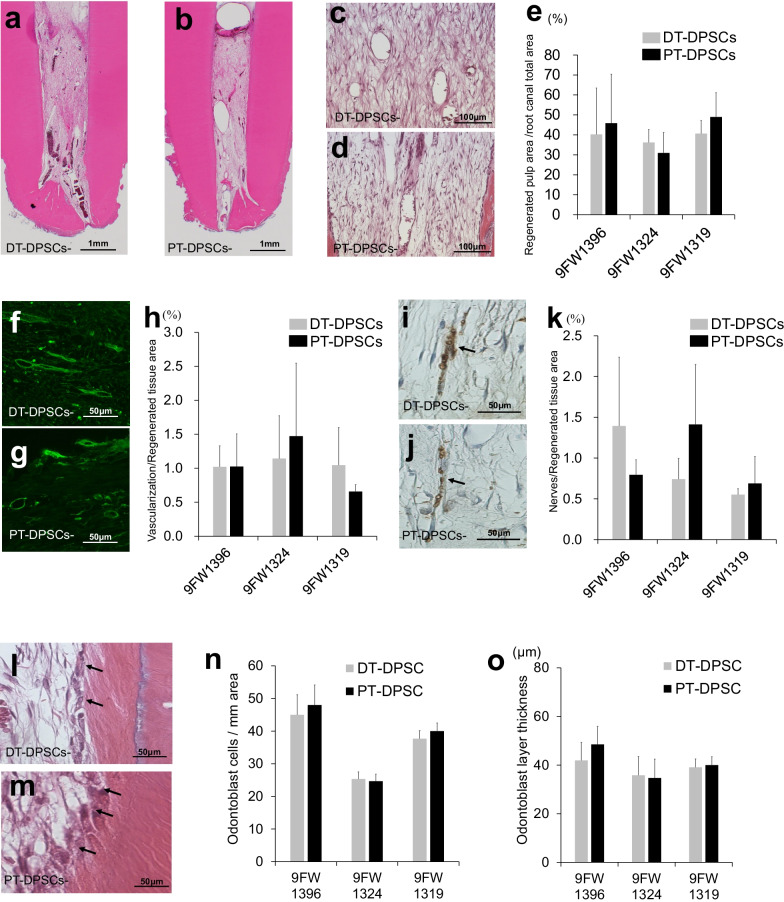
Complete pulp regeneration 4 weeks after autologous transplantation of DT-DPSCs compared with PT-DPSCs in the same individual dogs with pulpectomized teeth. **a**–**d** Regenerated pulp tissue with well-established vasculature. **f**, **g** Vasculogenesis stained with BS1-lectin. **i**, **j** Neurite extension shown by PGP9.5 immunostaining. **e**, **h**, **k** Statistical analyses of the regenerated pulp (**e**), vascularization (**h**), and reinnervation (**k**). **l**, **m** Osteodentin/tubular dentin-like mineralized tissue formation along the dentinal wall, odontoblast-like cells (arrows). **n**, **o** Statistical analyses of the odontoblast cells (**n**), odontoblast layer (**o**). **a**, **c**, **f**, **i**, **l** DT-DPSC transplants **b**, **d**, **g**, **j**, **m** PT-DPSC transplants. All the data are expressed as the mean ± standard deviation (*n* = 3)

The pulp regeneration efficacy of DT-DPSCs and PT-DPSCs was examined in pulpectomized dog teeth. Morphologically similar pulp tissue (Fig. 3a, b, c, d) and well-vascularized (Fig. 3f, g) and well-innervated (Fig. 3i, j) loose connective tissue were observed 4 weeks after DT-DPSCs and PT-DPSCs transplantation. Odontoblast-like cells were aligned along the dentinal wall in DT-DPSCs and PT-DPSCs transplants (Fig. 3l, m). There was no significant difference in the morphometric analysis (Table 2) and ratio of the regenerated pulp area (Fig. 3e) between the DT-DPSCs transplants and the PT-DPSCs transplants. Furthermore, no significant difference in neovascularization (Fig. 3h) or reinnervation (Fig. 3k) and odontoblasts cell number (Fig. 3n) or thickness (Fig. 3o) was demonstrated between the DT-DPSCs and PT-DPSCs transplants.

**Table 2:** Morphometric analysis of regenerated pulp tissue

	9FW 1396		9FW 1324		9FW 1319	
	DT-DPSC	PT-DPSC	DT-DPSC	PT-DPSC	DT-DPSC	PT-DPSC
Height (mm)	5.837 ± 1.4	6.257 ± 1.4	6.587 ± 0.6	5.019 ± 1.1	4.649 ± 0.6	5.459 ± 1.2
Width (mm)	1.607 ± 0.8	2.034 ± 0.4	1.438 ± 0.3	1.234 ± 0.6	1.211 ± 0.3	1.786 ± 0.4
Diameter(mm)	13.24 ± 2.5	14.2 ± 3.2	14.54 ± 1.9	11.11 ± 0.4	10.37 ± 0.8	12.32 ± 1.6
Area (mm^2^)	2.836 ± 0.8	3.95 ± 1.6	4.279 ± 1.7	2.962 ± 0.6	2.371 ± 0.6	2.776 ± 1.0

All data are expressed as the means ± SD (*n* = 3)

### No adverse effects

No adverse effects on appearance, clinical symptoms, food consumption, or body weight were observed in the toxicology assessment for 4 weeks after DT-DPSC transplantation. The values of serum and urine chemistry parameters were within normal ranges for 4 weeks (Additional file 3: Tables S1 & S2). No abnormalities were observed in any organs or tissues assessed by histopathological examination at 4 weeks. These results demonstrate that DT-DPSC transplantation might be safe for pulp regeneration.

## Discussion

There was no difference in the quality and quantity of regenerated pulp tissue, neovascularization and reinnervation between DT-DPSCs and PT-DPSCs transplants in our current in vivo study which indicate similar regenerative potential of DPSCs from permanent and deciduous teeth. Transplantation of atelocollagen only shows less amount of regenerated pulp tissue (Additional file 2: Fig. S2) [3] but large area of regenerated pulp tissue was observed after transplantation of pulp stem cells with G-CSF in atelocollagen scaffold in the present study. Besides pulp regeneration, odontoblast differentiation was also examined and compared, however, no significant difference was observed between DT-DPSCs and PT-DPSCs. These results indicated that deciduous teeth could be a potential source of DPSCs which may overcome the limited availability of pulp tissue or DPSCs from permanent teeth that hinders the autologous transplantation approach. For example, the isolation of stem cells from potentially discarded teeth, such as third molars, has certain limitations; for example, these teeth can be congenitally missing or impacted and sometimes function in occlusion. Furthermore, the reduction in pulp size or pulp stones that is often observed in elderly patients also limits DPSC isolation. Therefore, DPSCs isolated from human exfoliated deciduous teeth are important and convenient stem cell sources [8] because for child, there is an opportunity to isolate DPSCs from primary teeth when they naturally fall out and to store these cells in a cell bank for future autologous transplantation if it becomes necessary. Previous in vitro studies have already compared the stem cell properties and cytokine profiles of DPSCs and SHEDs [22, 23]. However, these cells were derived from different individuals. Thus, our current study was the first to compare stem cell properties in vitro and pulp regenerative potential in vivo of DPSCs and SHEDs from the same individuals.

Previous reports have characterized “stemness” as high expression of CXCR4, which indicates high migration potential [24], and high expression of the pluripotency marker OCT4 [25]. In the present study, a significantly higher number of CXCR4-positive cells and higher mRNA expression of *CXCR4* and *Oct 3/4* were observed in the DT-DPSC population than in the PT-DPSC population. These findings suggested a higher potential of DT-DPSCs for migration, self‐renewal and multilineage differentiation than PT-DPSCs. Another very important characteristic of mesenchymal stem cells is their high capacity for proliferation, and recent studies have demonstrated that SHEDs have much higher proliferation potential than DPSCs [9, 22]. We consistently found that DT-DPSCs had a higher proliferative capacity than PT-DPSCs and that DT-DPSC-derived CM significantly increased hPdLF proliferation compared to PT-DPSC-derived CM. In these respects, DT-DPSCs may be more suitable for storage in cell banks and clinical application in pulp regenerative therapy.

The migration of resident stem cells from the surrounding tissue is one of the critical mechanisms underlying pulp regenerative cell therapy, and G-CSF promotes the migration of transplanted DPSCs and resident stem cells [3]. In the present study, the DT-DPSC population had significantly higher percentage of G-CSFR-positive cells, as shown by flow cytometry, and higher migratory ability in the presence of G-CSF than PT-DPSCs; additionally, DT-DPSC-derived CM had stronger effects on hPdLF migration toward G-CSF than PT-DPSC-derived CM. Thus, G-CSF could enhance the migration of transplanted DT-DPSCs to a greater extent, and DT-DPSCs together with G-CSF might also enhance the migration of resident stem cells from the surrounding microenvironment more than PT-DPSCs.

Angiogenesis plays an important role in pulp regeneration after pulpectomy [26], and the angiogenic effects of SHEDs are stronger than those of DPSCs [27]. In the present in vitro study, no significant difference in *VEG*F mRNA expression but significant difference in protein expression was observed between DT-DPSCs and PT-DPSCs. This contrary expression is probably due to the various complex post-transcriptional mechanisms implicated in turning mRNA into a protein. DT-DPSC-derived CM induced significantly greater angiogenic tube formation in HUVECs after plating on Matrigel than PT-DPSC-derived CM, indicating a higher angiogenic effect of DT-DPSC-derived CM.

Reinnervation is also important for the functional recovery of regenerated pulp tissue, and *BDNF* and *NGF* are two of the major factors that regulate reinnervation [19]. A recent in vitro study performed a cytokine array and reported that BDNF and GDNF expression was upregulated in SHEDs and that NGF was highly expressed in DPSCs [23]. In this study, no difference in *NGF* and *GDNF* mRNA expression and significantly higher mRNA expression of *BDNF* in DT-DPSCs but similar protein expression of BDNF was demonstrated in DT-DPSCs compared to PT-DPSCs. These results might have occurred due to the different sources of the cells used in other studies, and the results of a quantitative analysis of cytokine production may not be directly related to cell properties. The similar effect of DT-DPSC-derived CM and PT-DPSC-derived CM on neurite extension in TGW cells observed in this study might have occurred due to the similar expression of BDNF in DT-DPSCs and PT-DPSCs culture supernatants. Further investigations are necessary to confirm the factors critical for neurodifferentiation that are released from DT-DPSCs and PT-DPSCs to enhance the reinnervation of regenerated pulp tissue.

Age-associated decreases in stemness, self-renewal, and regenerative potential of stem cells and age-associated increases in the expression of the senescence marker p16 have been described in DPSCs [28]. Our present study revealed significantly lower mRNA expression of *p16* in DT-DPSCs than in PT-DPSCs. Thus, decreased expression of senescence markers may improve the therapeutic effects of stem cells, and DT-DPSCs may be considered as cells with reduced senescence characteristics.

Anti-inflammatory properties are important factors that enhance the therapeutic potential of DPSCs in pulp regenerative cell therapy [29]. DT-DPSC-derived CM and PT-DPSC-derived CM exerted similar immunosuppressive effects on canine PBMCs, indicating that DT-DPSCs are a potential substitute for PT-DPSCs.

In the present in vivo study, the pulp regenerative potential, angiogenesis, reinnervation and odontogenic potential of DT-DPSCs were similar to those of PT-DPSCs isolated from the same individuals, while higher expression of some stem cell markers, higher angiogenic, migration potential and identical neurogenic potential were observed in vitro. These results might have occurred due to the young age (1 year) of the experimental dogs, which have resident stem cells with high regenerative potential and a surrounding microenvironment that promotes pulp regeneration. Transplanted stem cells release trophic factors to promote tissue regeneration rather than to differentiate into properly functional cells themselves [30]. However, the levels of trophic factors derived from transplanted DPSCs that enhance resident stem cell migration, cell survival, anti-inflammatory mechanisms, pulp regeneration, angiogenesis and reinnervation might exceed the levels required for the establishment of an optimal microenvironment for pulp regeneration in young dogs. Thus, further study in aged dogs may be needed to reach proper conclusions.

DT-DPSCs are easily accessible with the patient's safety assured, posing less ethical issues and dental pulp of deciduous teeth is present before birth and is maintained before eruption of permanent teeth, this period is characterized by maintenance of an active niche rich in stem cells, which are not yet heavily affected by the intensifying effect of genetic and/or environmental factor [31]. Recently, cell banking and preservation of dental stem cells have become a promising and advanced scientific topic not only in tooth regeneration but also in other regenerative medical fields [32, 33]. To overcome the limited availability of discarded permanent teeth and inadequate sources of DPSCs from older donors and donors with systemic diseases, including diabetes and rheumatoid arthritis, SHEDs are the best candidates for cell banking [34]. Our present results demonstrated significantly lower expression of *p16* in DT-DPSCs than in PT-DPSCs, indicating the stability of the stem cell phenotype after prolonged cell culture, rendering these cells suitable for cell banking.

## Conclusions

In the present study, DT-DPSC shows higher expression of some stem cell markers, angiogenic potential and identical neurogenic potential in vitro and in vivo there was no difference in pulp regenerative, angiogenic and reinnervation potential between DT-DPSCs and PT-DPSCs derived from the same individuals, indicating that DT-DPSCs are a potential substitute for PT-DPSCs as a stem cell source.

## Supplementary Information

**Additional file 1: Fig S1**. Flow cytometry analysis of MSCs-specific cell surface markers. Representative histograms for cell surface markers in DT-DPSCs and PT-DPSCs. Each type of DPSCs was analyzed at passage 4 using a flow cytometer (*n* = 3).

**Additional file 2: Fig S2**. Effect of atelocollagen only on pulp tissue regeneration Representative image of hematoxylin and eosin staining of the regenerated pulp tissue after transplantation of atelocollagen without dental pulp stem cells.

**Additional file 3: Table S1 & S2**. Safety evaluation by blood chemistry examinations and urinalysis at 4 weeks after the transplantation of DT-DPSCs. **Table S3**. Canine Primers used for Real-Time RT-PCR.

## Data Availability

All data generated or analyzed during this study are included in this published article.

## Abbreviations

PT-DPSCs: Dental pulp stem cells isolated from permanent teeth
DT-DPSCs: Dental pulp stem cells isolated from deciduous teeth
G-CSF: Granulocyte colony-stimulating factor
CXCR4: C-X-C motif chemokine receptor 4
G-CSFR: Granulocyte colony-stimulating factor receptor
Oct3/4: Octamer-binding transcription factor 4
BDNF: Brain-derived neurotrophic factor
MSCs: Mesenchymal stem cells
DPSCs: Dental pulp stem cell
SHEDs: Stem cells from human exfoliated deciduous teeth
HIF-1α: Hypoxia Inducible Factor 1
CM: Conditioned medium
VEGF: Vascular endothelial growth factor
hPdLF: Human periodontal ligament fibroblasts
PBMCs: Peripheral blood mononuclear cells
STAT3: Signal transducer and activator of transcription 3
GM-CSF: Granulocyte monocyte colony-stimulating factor
GDNF: Glial cell-derived neurotrophic factor
NGF: Nerve growth factor
TRH-DE: Thyrotropin releasing hormone degrading enzyme
IL-1β: Interleukin1 beta
IL-8: Interleukin 8
ELISA: Enzyme-linked immunosorbent assay
