# Supplementary figures and images for: Biological characteristics and pulp regeneration potential of stem cells from canine deciduous teeth compared with those of permanent teeth
Source: Stem Cell Res Ther. 2022 Sep 2;13:439. doi: 10.1186/s13287-022-03124-3 (PMC9438285; doi:10.1186/s13287-022-03124-3)

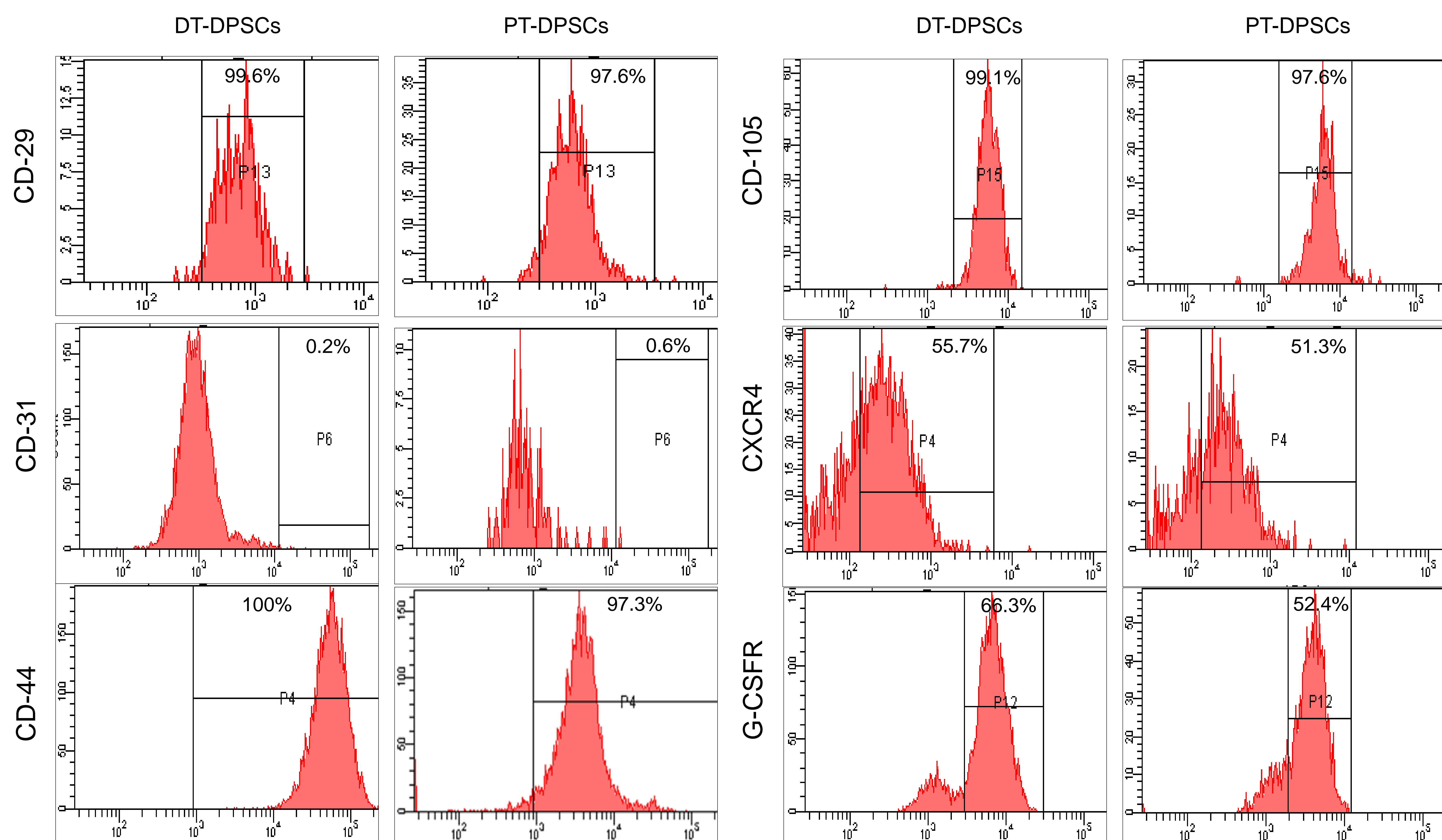

Supplementary Figure 1

Supplement: Supplementary file 1 — Additional file 1: Fig S1. Flow cytometry analysis of MSCs-specific cell surface markers. Representative histograms for cell surface markers in DT-DPSCs and PT-DPSCs. Each type of DPSCs was analyzed at passage 4 using a flow cytometer (n = 3). [file 13287_2022_3124_MOESM1_ESM.pdf]

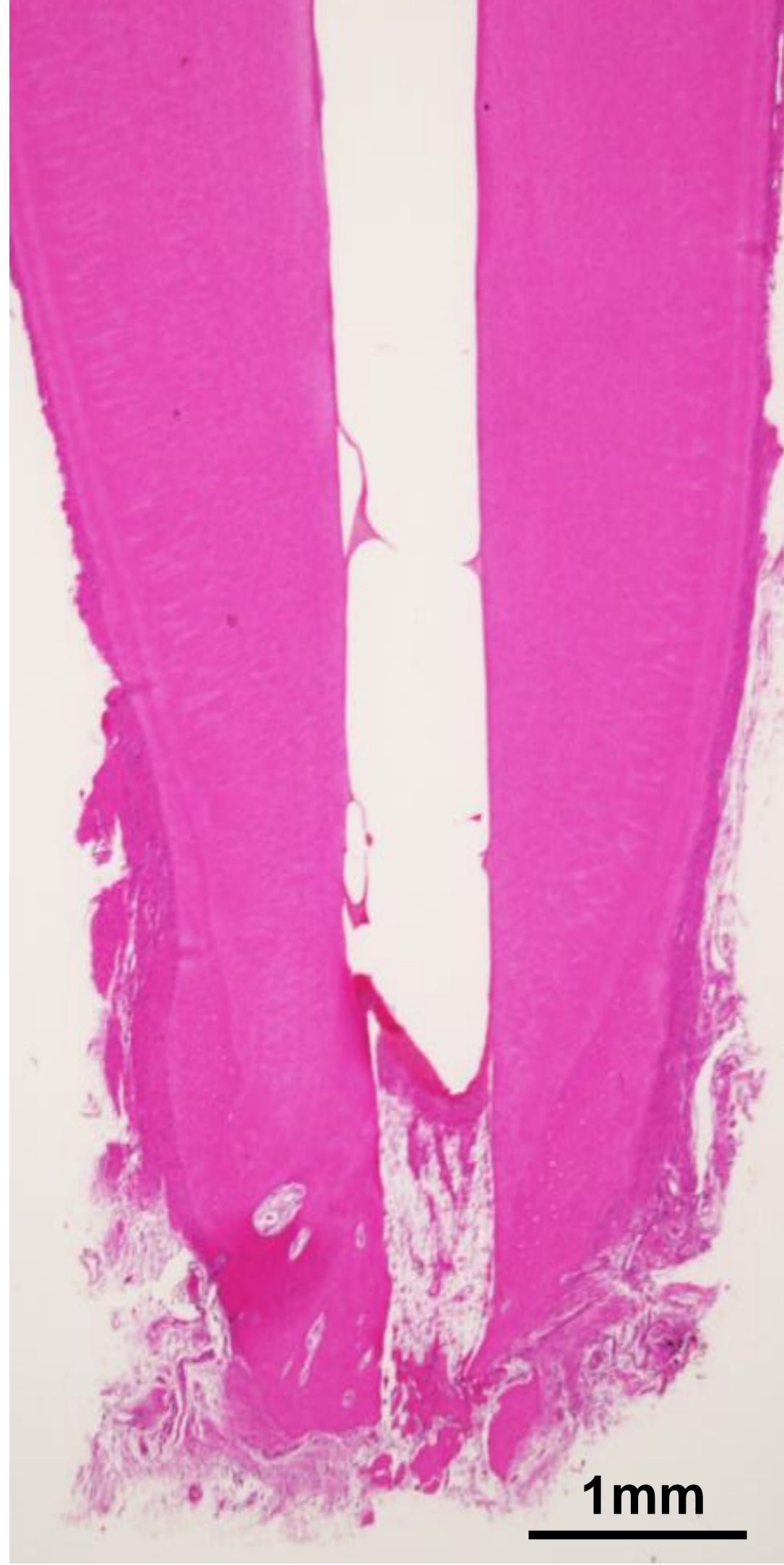

Supplementary Figure 2

Supplement: Supplementary file 2 — Additional file 2: Fig S2. Effect of atelocollagen only on pulp tissue regeneration Representative image of hematoxylin and eosin staining of the regenerated pulp tissue after transplantation of atelocollagen without dental pulp stem cells. [file 13287_2022_3124_MOESM2_ESM.pdf]
