# Supplementary material for: Biological characteristics and pulp regeneration potential of stem cells from canine deciduous teeth compared with those of permanent teeth
Source: Stem Cell Res Ther. 2022 Sep 2;13:439. doi: 10.1186/s13287-022-03124-3 (PMC9438285; doi:10.1186/s13287-022-03124-3)
Supplement: Supplementary file 3 — Additional file 3: Table S1 & S2. Safety evaluation by blood chemistry examinations and urinalysis at 4 weeks after the transplantation of DT-DPSCs. Table S3. Canine Primers used for Real-Time RT-PCR. [file 13287_2022_3124_MOESM3_ESM.pdf]

Supplementary table 1

Safety evaluation of by blood test and blood chemistry examinations at 4 weeks after the transplantation of DT-DPSCs

Blood Chemistry examination

Individual number	AST (IU/L)	ALT (IU/L)	Albumin (g/dL)	Total Protein (g/dL)	Bilirubin (mg/dL)	Creatinine (mg/dL)	Urea Nitrogen (mg/dL)	A/G	Na (mEq/L)	Cl (mEq/L)	K (mEq/L)	CRP (mg/dL)
9FW1319	64	34	3.2	6.4	0.0	0.52	18.0	1.00	151	102	4.4	<0.05
9FW1324	55	38	2.8	6.0	0.0	0.61	21.9	0.88	152	109	5.0	<0.05
9FW1396	76	35	3.4	6.6	0.0	0.51	20.1	1.06	148	101	4.7	<0.05
average	65.0	35.7	3.1	6.3	0.0	0.55	20.0	0.98	150.3	104.0	4.7	<0.05
S.D.	8.60	1.70	0.25	0.25	0.00	0.04	1.59	0.07	1.70	3.56	0.24	-
Normal range	17-78	20.8-58.8	2.67-3.43	4.81-6.17	0.004-0.08	0.521-0.825	4.8-31.4	0.899-1.659	140-152	102-117	4.07-5.07	<1.0

Notes AST: Aspartat transminase
ALT: Alanine transminase

Supplementary table 2

Safety evaluation of by urinalysis at 4 weeks after the transplantation of DT-DPSCs

Urinalysis

Individual number	Specific Gravity	pH	Urobillinogen	Birilbin	Keton	Color
	28days	28days	28days	28days	28days	28days
9FW1319	1.007	8	+−	-	-	Normal
9FW1324	1.005	8	+−	-	-	Normal
9FW1396	1.007	8	+−	-	-	Normal
average	1.0	8.0	+−	-	-	-
S.D.	0.00	0.00	/	/	/	/
Normal range	1.008-1.05	5.5-8.5	+−	-	-	Normal

Supplementary table 3

Canine Primers for Real-Time RT-PCR

Gene	Forward primer sequence (5'-3')	Reverse primer sequence (5'-3')
HIF-1a	ACTGATGACCAACAACTTGAGG	TTTGGAGTTTCAGAAGCAGGTA
Oct3/4	ACGATCAAGCAGTGAC	GAGGGACTGAGGAGTG
STAT3	GTGGTGACGGAGAAGCAACA	TTCTGTCTGGTCACCGACTG
CXCR4	ACTCCATGAAGGAACC	TGCCCACTATGCCAGT
NANOG	GAATAACCCGAATTGG	AGCGATTCCTCTTCAC
GM-CSF	CTCACCAGCCTCAAGAATCC	GGACTGGTCTGCCTCACTTC
VEGF	CTACCTCCACCATGCCAAGT	ACGCAGGATGGCTTGAAGAT
BDNF	GTTGGCCGACACTTTTGAAC	CCTCATCGACATGTTTGCAG
NGF	CAACAGGACTCACAGGAGCA	ATGTTCACCTCTCCCAGCAC
GDNF	GCCGAGCAGTGACTCAAAC	TCTCGGGTGACCTTTTCAG
TRH-DE	CAACAAGCACTGTCACCAAC	ATTCCCAGACATCCTCATCC
IDO	GGAAAGGCAACTCCAAACTG	CCCAGCAGAATGTCAAAGC
p16	CGGAAGGTCACGCAGACAGC	GCAGGGAAGAGTTGGGTTGGGT
p 21	ACCTCTCAGGGCCGAAAAC	TAGGGCTTCCTCTTGGAGAA
IL-1β	CAAGAGTCTGAGGCATTTC	GGTATTTGTGGCTTATGTCC
IL-8	ACACTCCACACCTTCCAT	CTTTTGTACCCATTTTTCC
β-actin	AAGTACCCCATTGAGCACGG	ATCACGATGCCAGTGGTGCG
